# Synthesis and characterization of Ethiopian kaolin for the removal of basic yellow (BY 28) dye from aqueous solution as a potential adsorbent

**DOI:** 10.1016/j.heliyon.2020.e04975

**Published:** 2020-09-19

**Authors:** Tadele Assefa Aragaw, Fikiru Temesgen Angerasa

**Affiliations:** Faculty of Chemical and Food Engineering, Bahir Dar Institute of Technology, Bahir Dar University, Bahir Dar, Ethiopia

**Keywords:** Materials science, Chemical engineering, Dye wastewater, Kaolin, Adsorption, BY 28 dye, Adsorbent synthesis, Characterization

## Abstract

In the present research, the kaolin adsorbents (beneficiated, raw powder, and calcined) were prepared from Ethiopian natural kaolin through mechanical, wet, and thermal processes. The geochemical and surface properties of kaolin adsorbent were characterized using FTIR, SEM/EDS, XRD, and XRF. In the batch experiment, basic operation parameters (initial dye concentrations, pH, temperature, contact time, and adsorbent dosage) were examined. Percentage removal efficiency basic yellow 28 (BY28) dye were recorded as 94.79%, 92.08%, and 87.08% onto beneficiated, raw, and calcined kaolin absorbents, respectively at an initial dye concentration of 20 mg/L, solution pH of 9, the temperature of 30 °C°C, and contact time of 60 min and adsorbent dosage of 1g/100L. The molar ratio of SiO_2_/Al_2_O_3_ was recorded as 2.911 Percent mass composition of Ethiopian kaolin which is higher than the expected pure kaolinite standard which allows us to classify the kaolin clay as a siliceous one. The calculated values of $\Delta G^{0}$ for beneficiated adsorbent are -1.243, 1.576, and 4.396 kJ/mol at 303.15, 323.15, and 343.15 K, respectively for 20 mg/L of dye concentration and solution pH of 9, suggests that the thermodynamic behavior at lowest temperature is more feasible and spontaneous as compared with the higher temperature one. A similar fashion was calculated for raw and calcined adsorbents. The negative values of ΔH^o^ and ΔS° suggest that the adsorption phenomenon is exothermic and the adsorbate molecules are organized on the solid phase in a more disordered fashion than the liquid phase. The pseudo-first-order and pseudo-second-order models have been used to describe the kinetics in the adsorption processes. The Pseudo-second-order model has been fitted for the BY 28 dye adsorption in the studied concentration range. The adsorption of BY 28 dye for raw and calcined adsorbents follows the Langmuir isotherm and the Freundlich isotherm fitted for the beneficiated adsorbent. The amount of BY28 dye taken up by beneficiated, raw, and calcined kaolin adsorbents was found as 1.896, 1.842, and 1.742 mg/g, respectively at a contact time of 1.0 h, the adsorbent dosage of 1.0 g, initial dye concentration = 20 mg/L and solution pH = 9 at 30 °C. The results found that these raw and prepared local kaolin adsorbents have a capacity as low-cost alternatives for the removal of dyes in industrial wastewater.

## 1 Introduction

Currently, industrial manufacturing companies are increasing worldwide due to population growth, and the wastewater produced these industries have been discharging it the natural water bodies without proper treatment. Wastewater from the industrial sector is mainly characterized by organic compounds, inorganic compounds, heavy metals, and highly colored. The textile dyeing effluent has toxic properties and changes the water body's characteristics [1]. Particularly, azo dyes and its degraded aromatic amines are highly toxic, mutagenic, and carcinogenic. Also, they can decrease light penetration and photosynthetic activity in the water system, ultimately causing oxygen deficiency and limiting downstream beneficial uses, such as recreation, drinking water, and irrigation [2]. Some methods were used for dye removal from industrial wastewater like filtration [3], flotation [4], adsorption [1, 5], and photocatalysis [6, 7]. Adsorption has been recognized as a potential technology for the removal of dyes from wastewater [1]. Different types of adsorbents can be prepared from different raw materials to adsorb fine particles, molecules, or ions from solution [8]. A clay mineral, kaolin is composed of kaolinite material (Al_2_Si_2_O_5_ (OH)_4_), which has been widely used in a variety of technological applications. Kaolin clay is a promising adsorbent and attracts attention due to the alternative low-cost, eco-friendly, and highly abundant. Specifically, kaolin from different country has been investigated to remove different dyes form aqueous solution like malachite green [9], basic red 46 and direct blue 85 [10], methyl orange [11], violet 5Rand acid blue 25 [12], methylene blue, crystal violet and congo red [13], methylene blue [14, 15], methyl violet 10B [16], basic yellow 28 dye [17], brilliant green and crystal violet [18], reactive red 120 [19]. The other important thing is that natural adsorbents, such zeolite, and clay-based (bentonite, kaolin, and montmorillonite) adsorbents, in addition to good rheological and adsorptive properties, are cost-effective, eco-friendly, widely used owing to their simplicity, and good efficiency and their simple regeneration techniques [20]. Even though activated carbon is the most effective for dye adsorption, its use is uneconomical because of the regeneration cost is high as compared with clay-based adsorbents [21]. Several regeneration techniques (Fenton oxidation, supercritical extraction, thermal degradation, etc....) is a critical aspect to rouse the adsorption efficiency of the spent adsorbent for contaminant removal. In a sense of low cost, the adsorbent depends on various factors, such as the availability of materials, and source (natural-based, waste biomass, by-products, or synthesized products), the ease of preparation, recyclability, and country of production [22]. Ethiopian has enormous minerals including kaolin clay as per the geological survey of Ethiopia, unpublished report. Kaolin clay was surveyed and found across different regions but is not used as a commercial raw material for industries as well as for water and wastewater purification. Thus, this research work is also important to examine the geochemical properties of Ethiopian kaolin which will have evidence to the sector as a preliminary investigation about the grade (quality) of the clay.

This study anticipates the removal of basic yellow dye with raw, wet, and thermally treated Ethiopian kaolin as a potential and cost-effective adsorbent. Raw and treated adsorbent characteristics were discussed using FTIR, SEM, XRD, and XRF. The batch experiments with different parameters, such as solution pH, adsorption temperature, mixing time, adsorbent load, and the concentration of BY28 dye were carried out and optimized. The adsorption processes such as adsorption isotherms with Freundlich and Langmuir model, adsorption kinetics with pseudo-first and second-order kinetic models, and thermodynamics properties were well examined.

## 2 Materials and methods

### 2.1 Chemicals and equipment

Different mechanical size reduction equipment (jaw crusher and disk mill, sieves); classical thermal equipment (Hot air oven and muffle furnace) were used for adsorbent preparation. Also, analytical equipment and glassware such as analytical balance, pH meter, and centrifuge, hot plate with a magnetic stirrer, measuring cylinder, test tubes, and pipette were frequently used for batch adsorption experiments. Basic yellow 28 (BY28) dye, concentrated sulphuric acid, sodium hydroxide was used for synthetic dye solution preparation; and pH adjustment during the batch experiment. The powder BY28 dye and the lab grade chemicals, such as sodium hydroxide, Hydrochloric acid, and potassium bromide were used without any purification.

### 2.2 Adsorbent and adsorbate preparation

#### 2.2.1 Sample collection and beneficiation of kaolin

Natural kaolin was collected from the local area, Debre Tabor Town, Amhara Region, Ethiopia. Collected natural kaolin was subjected to size reduction (crushed, milled, and screened) to produce fine powder using a jaw crusher (BB50), disc mill (Pulvisette 13), and Standard sieves (ISO9001) for 75μm. the fine kaolin powder, of 75μm, was used for batch experiment and characterization as a ‘‘raw kaolin adsorbent’’ without further treatment. After mechanical size reduction of kaolin powder with the particle size of 75μm, it was subjected to wet treatment (beneficiation) using distilled water in the conical shape glassware of 1L capacity. The beneficiation process was undergone by shaking for 24 h in such a way that the powder fully disperses and make suspensions. The beneficiation purification techniques as a pretreatment for clay materials are crucial for the removal of different suspended and/or soluble salts, organics contained as well as coarse particles to make good adsorbent surfaces. After 24 h beneficiation processes, the suspension was allowed to settle for 1 h to separate the fine and coarse particles. After 1 h sedimentation time, the suspension shows that three different layers (turbid supernatant, slurry at the middle, and a coarse particle at the bottom). The middle, slurry, were dried at 70 °C to remove absorbed water using hot air oven. The dried beneficiated cake-like product was ground, milled, and sieved for less than 75 μm. The beneficiated, dried, and sieved product was used as a ‘‘beneficiated adsorbent’’ for batch adsorption experiment and characterization.

#### 2.2.2 Thermal treatment

Mechanically size reduced powder raw kaolin (75 μm), not beneficiated kaolin, was used for thermal activation (calcination). Metakaolin can be produced with a range of calcination temperatures depending on the age, and formation of kaolin clay. Commonly, the dehydroxylation process can occur from 550 °C to 850 °C to produce disordered metakaolin [23]. The wide range of calcination is due to a complex amorphous structure that can maintain because of the heaping of the nature of hexagonal layers [24]. The thermogravimetric analysis of Ethiopian kaolin where the samples are taken was determined from my previous work and dehydroxylation occur up to 700 °C [25]. Thus, powder raw kaolin (75 μm) was calcined at 700 °C for 3 h using Muffle Furnace (Nabertherm B180) and used for as a ‘‘calcined adsorbent’’ for adsorption study and characterization.

### 2.3 Preparation of stock solution and standard calibration

**Figure 1:**
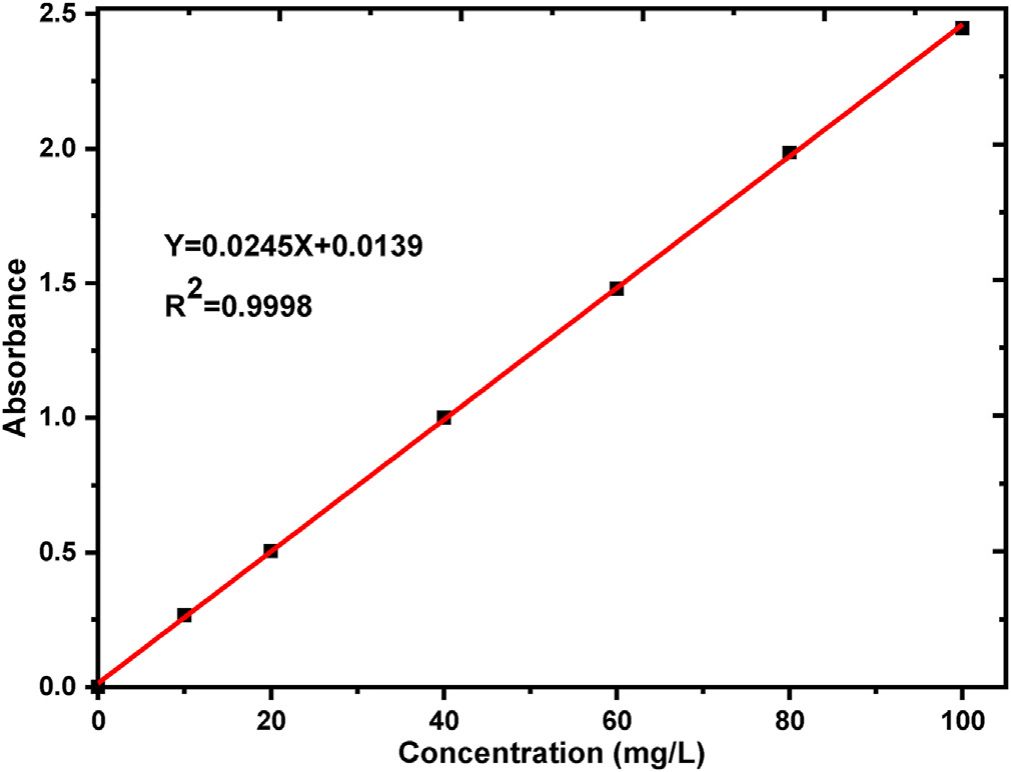
Calibration curve for the standard solution of BY 28 Dye.

Basic yellow 28 (BY 28) dye, a modal dye in the textile industry, was collected from Bahir Dar Textile Share Company, Ethiopia. The physical state of basic yellow dye is a powder with a chemical formula of C_21_H_27_N_3_O_5_S and molecular weights of 433.52 g/mol. The stock solution (500 mg/L) of BY28 dye was prepared by dissolving 0.5 g of in 1 L of distilled water and; working and standard solutions (10, 20, 40, 60, 80, and 100 mg/l) by dilution of the stock solution. The UV/Vis spectra scanning with a range of 200–700 nm were conducted to determine the lambda maximum (λ_max_) with the corresponding absorbance of dye solution using a UV/VIS spectrophotometer (PerkinElmer Lambda 35) and recorded at 438 nm. For each batch adsorption experiment, the final dye concentration was calculated based on the linear calibration curve as shown in Figure 1.

### 2.4 Experimental design and description

With a Pyrex laboratory flask of 250 ml, the desired concentration of dye solutions (100 ml) and 1 g of adsorbent (raw beneficiated and calcined kaolin adsorbents) were mixed and stirred on a digital hot plate at 200 revolutions per minute (rpm). Frequently, the pH of the solution was adjusted with 1M HCl or 1M NaOH before adding the adsorbents into the dye solution. The batch adsorption experiments were performed with a predesigned range of operating parameters. The 20, 40, and 60 mg/L of the initial BY28 dye concentration; 30, 50 and 70 °C of the adsorption temperature; 3, 7, and 9 of the solution pH; 0.1, 0.5, 1, 1.5, and 2 g of the adsorbent dosage; and 20, 40, 60, 80 and 100 min of the mixing time were performed. The adsorption experiments for the three adsorbents at an optimized rpm (200) were conducted. The supernatants of the solutions after each adsorption experiment were taken and centrifuge, at 4000 rpm for 10 min, using a rotary centrifuge machine (SIGMA 3–18 KS). The subsequent absorbance of residue was determined using a UV/Vis spectrophotometer (PerkinElmer Lambda 35). The final BY28 dye concentrations were calculated as per the calibration curves shown in Figure 1. The removal efficiency of the BY 28 dye was calculated as from Eq. (1) [26]. The equilibrium (qe, mg/g) and at any time (qt, mg/g) concentration of BY28 dye in the solid phase in the adsorption phenomenon were calculated using Eqs. (2) and (3), respectively [27].

(1) $$Removal\;Efficiency\;\left(\%\right)=\frac{\left(C_{o}-C_{t}\right)}{C_{o}}\times 100$$

(2) $$q_{e}=\frac{V\left(C_{o}-C_{e}\right)}{m}$$

(3) $$q_{t}=\frac{V\left(C_{o}-C_{t}\right)}{m}$$

Where, C_o_ is the initial dye concentration (mg/L), C_e_ is dye concentration in the liquid phase at the equilibrium (mg/L), C_t_ is the dye concentration in the liquid phase at any time (mg/L), m is the amount of adsorbent (g) and V is the volume of solution (L).

The point at which the y-axis with the measured values of the x -coordinate where the zero value of ΔpH is an indication of the point of zero charges [28]. The point of zero charges (pHpzc) of the three adsorbents was regulated using KCl, NaOH, and HCl solutions. Thus, firstly the solution of 50 ml was prepared in six beakers and the initial pH (pHi) values were adjusted in the range of 2–12 by adding 0.1M HCl and 0.1 NaOH solution and 0.1 M KCl as supporting electrolyte solution were added in each beaker. The adsorbents of 1 g were added in the beaker and allowed for 24 h. After equilibration time, the filtration techniques of the turbid suspension solutions were carried out, and final pH values (pH f) of the filtrates were measured. The (pHpzc) for the raw, calcined, and beneficiated kaolin adsorbents were plotted pHi-pH f versus pHi.

### 2.5 Adsorbent characterization

Fourier transform infrared, FTIR, (ILC38B6PD7) spectrophotometer was used for the investigations of bending vibration bond stretching spectra's for raw, calcined, and beneficiated kaolin adsorbents as well as before and after dye adsorption with a range of 400–4000 cm^−1^. Pelletization was performed with a reagent grade KBr standard; grounded uniformly using mortar and pestle at 1 to 100 KBr to sample ration. The surface morphology and elemental distribution of the raw, calcined, and beneficiated kaolin adsorbents were examined using a scanning electron-microscope with the corresponding Energy Dispersion X-ray spectroscope using Inspect™ Scanning Electron Microscope, SEM (INSPECT F50) with different magnification and scale bar. The crystalline structure was identified for raw beneficiated and calcined kaolin adsorbents and the diffraction patterns were measured using an x-ray diffractometer, XRD (MIN 3740) with CoK radiation. XRD diffractograms were obtained between the X-ray intensity on the ordinate and the Bragg angle, θ, on the abscissa were performed on a θ–2θ angle ranging from 4.99^0^ to 90.0^0^ through continuous scanning at a step size of 0.0131303° with a counting time per step of 125.97 at a 40 Kv Generator voltage and tube current 40 mA K-Alpha1 wavelength: 1.78901; K-Alpha 2 wavelength 1.7929 at K-Alpha 2/K-Alpha 1 ratio of 0.5. Whole rocky by fusion/x-ray fluorescence (XRF) with a specific applied method of ME-XRF26 together with their Loss on ignition (LOG-24) by furnace were used for the composition of the chemical oxides for raw, beneficiated, and calcined kaolin adsorbents. Both XRD and XRF analyses were conducted from Australia and Ireland through Australian laboratory service (ALS) at a division of geochemistry respectively.

### 2.6 Adsorption phenomenon study

#### 2.6.1 Isotherm models

Two basic and simple adsorption isotherm models, Langmuir and Freundlich, were employed to study the relationship between BY 28 dye ions adsorbed on the raw, beneficiated, and calcined kaolin adsorbents and residuals in the dye solution. The Langmuir and Freundlich model was calculated with Eqs. (4) and (5), respectively, and the shape of the isotherms ($R_{L})$ were calculated with Eq. (6) [29, 30].

(4) $$\frac{C_{e}}{q_{e}}=\frac{C_{e}}{q_{m}}+\frac{1}{q_{m}K_{L}}$$

(5) $$logq_{e}=\mathrm{logkf}+\frac{1}{n}logC_{e}$$

(6) $$R_{L}=\frac{1}{1+K_{L}C_{o}}$$

Where q_m_ is sorption capacity (mg/g), K_L_ is sorption energy (L/g), Kf, and n are the Freundlich constants. If n = 1, n > 1, and n < 1, then the sorption process would be the linear, physical, or chemical in its nature, respectively. Where K_L_ is the Langmuir constant related to the energy of adsorption (L/mg) and Co is the highest initial dye concentration (mg/L).

#### 2.6.2 Kinetics model

The basic two adsorption kinetics model (pseudo-first and second-order) was applied for batch adsorption experiments to determine the adsorption mechanism for the corresponding allowed time with governing Eqs. (7) and (8), respectively [31, 32].

(7) $$\log\left(Qe-q_{t}\right)=\log q_{e}-\frac{k_{1}}{2.303}t$$

(8) $$\frac{t}{q_{t}}=\;\frac{1}{k_{2}q_{e}^{2}}+\;\frac{1}{q_{e}}t$$

Where, $q_{e}$ and $q_{t}$ are the amounts of adsorbate adsorbed (mg.g^−1^) at equilibrium and at any time t, respectively. As well as $k_{1}$ (min^−1^), $k_{2}$ (g.mg^−1^min^−1^) are the pseudo-first and second-order rate constants, respectively.

#### 2.6.3 Thermodynamic behaviors

The value of standard change Gibbs free energy, enthalpy, and entropy in the adsorption process was calculated with Eqs. (9) and (10) with the adsorption temperature [25]. Whether the thermodynamic process is exothermic or exothermic in the adsorption process can give evidence for the solid-liquid interactions, and the ratio of the equilibrium concentration of the dye solution was estimated with Eq. (11) [33, 34, 35].

(9) $$\Delta G^{0}=\Delta H^{0}-T\Delta S^{0}$$

(10) lnKc=−ΔG0RT=ΔS0R−ΔH0RT

(11) Kc=qeCe

Where ΔG^0^ = standard change free Gibbs energy (kJ mol^−1^), $\Delta {\text{H}}^{0}$ = standard change enthalpy (J mol^−1^), $\Delta {\text{S}}^{0}$ = standard change entropy (J mol^−1^K^−1^), and R = universal gas constant (8.314 J mol^−1^K^−1^). Kc is the ratio of the equilibrium concentration of adsorbate (q_e_) loaded to equilibrium concentration in solution (Ce).

### 2.7 Regeneration and reuse of the adsorbent

To be evident the sustainability of the raw and prepared adsorbents, evaluating the regeneration, and reuse potential are important. Thus, BY28 dye batch experiments were conducted by treating a solution containing 20 mg/L BY28 dye with 1.0 g of the three adsorbents at initial pH of 9 for 60 min at room temperature. After agitation, mixtures were allowed for settling and centrifuge. The suspension like residues was washed multiple time with deionized water and oven-dried for 14 h at 70 °C for the subsequent experiments. Then, the dried samples were milled and sieved using miller and standard sieve (ISO9001) to pass through less than 75 μm sieve, weighed, and then regenerated using 100 mL of 0.01 M K_2_CO_3_ by agitating the mixture for 60 min. Many studies on the sorption-desorption have been dealt with alkali carbonates, such as Na_2_CO_3_ and K_2_CO_3_. The present authors use potassium carbonate which is allowed to maintain a good capacity for many sorption-desorption cycles. But, sodium carbonate drastically decreases the sorption capacities as a function of the number of sorption-desorption cycles due to a sintering effect of salt-salt and salt with the adsorbents [36]. The binary (K_2_CO_3_/LiCO_3_, Na_2_CO_3_/LiCO_3_) eutectic mixtures are by far is good for sorption capacities as functions of number reuse [37]. After regeneration, the three adsorbent types were used for BY28 dye removal. The regeneration-reuse cycle was continued up to the 7th cycle.

## 3 Result and discussion

### 3.1 Analysis of FTIR

**Figure 2:**
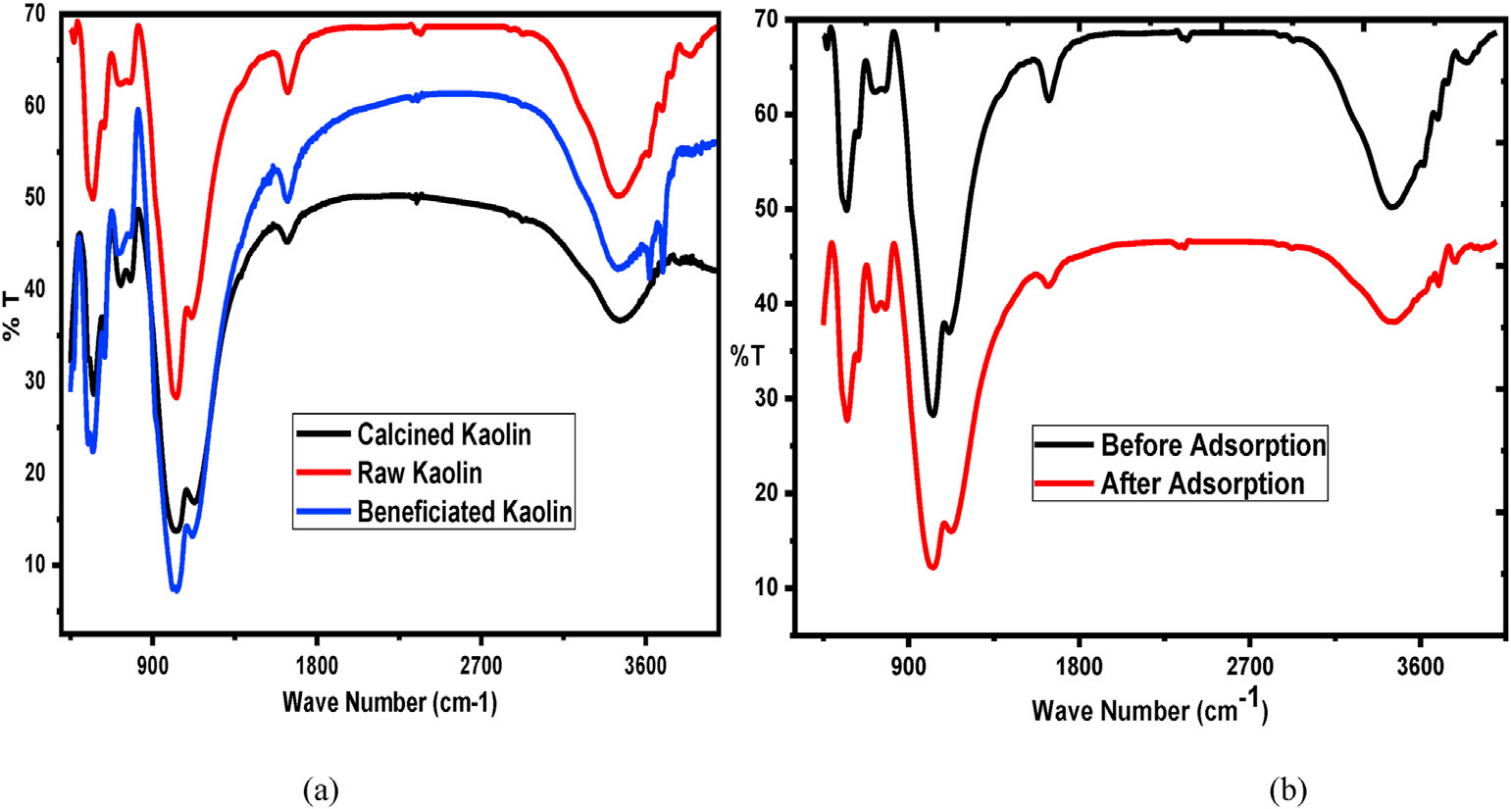
FTIR analysis of kaolin adsorbents (a) before adsorption of the three adsorbents (b) after adsorption.

The FTIR analyses of raw, calcined, and beneficiated kaolin adsorbents, and after adsorptions are depicted in Figure 2 and Table 1. The tabulated wavenumbers with the corresponding band assignments as shown in Table 1 include the differences before adsorptions of raw kaolin adsorbent with after adsorption. The broadband around 3468 cm^−1^ represents hydroxyl stretching vibration (Al-OH or Si-OH) and characteristics band of kaolin. A small peak around 3821cm^−1^ in the raw and beneficiated kaolin is the inner surface hydroxyls attached to Al or O which can form the bonds between octahedral and tetrahedral sheets. Similar results were reported by [38]. The inner surface hydroxyl group which is used as hydrogen bonding for the two layers in the kaolin structure were disappeared suggested that dehydroxylation occurred during the thermal treatments or raw kaolin. A similar result was reported in the study of Ethiopia kaolin characterization and zeolite synthesis from it [39]. Peaks around 1114 cm^−1^ and 1023 cm^−1^ are corresponding with silicon monoxide (Si–O) and Al-O stretching vibration related to the presence of quartz and di-silicon oxide (Si-O-Si) symmetric stretch stretching vibration respectively indicates that silicon oxide is contained dominantly in the studied kaolin [40]. The small peak at 2370 cm^−1^ attributes the deformation of hydroxyl from the water molecule [41]. The peaks at 2922 cm^−1^ from the raw kaolin attribute aliphatic hydrocarbon (CH) stretching which indicates that there is an organic impurity, but disappears from the calcined one. The small peak at 785 cm−1 attributes metal impurity bonded with aluminum and hydroxyl (Al-Mg-OH) vibration [42] Sharp and clear peak around 584 cm^−1^ corresponds with Si-O-Al bending vibration indicate that aluminum oxide in also contained next ton silicon oxide. Small intensity peaks at 1635 cm^−1^ correspond to water molecules adsorbed on the kaolin surface. At this peak, comparatively the intensity for raw kaolin is higher than the calcined one due to moistures were removed during calcination. As can be observed from Figure 2 (b), the bending shape especially at 3468 cm^−1^, 1635 cm^−1,^ and 1114 cm^−1^ stretching vibrations at the surface of the adsorbent before adsorption is greater than after adsorption. This suggests that adsorbent surface after adsorption were loaded by the adsorbate molecules. Thus, the percentage of transmittance has been decreased as compared with before adsorption of the adsorbent.

**Table 1:** FTIR spectra bands of raw, calcined, and beneficiated kaolin with the corresponding assignments.

Wavenumber (cm−1)				Differences (raw with after)	Assignments	Reference
Before			After			
Raw	Calcined	Beneficiated	Raw			
3695	–	3693	3696	+1	–	[43]
3620	–	3616	–	–	OH stretching of water	[43]
3468	3468	3469	3469	+1	OH (Al-OH or Si-OH) stretching	[44]
2922	–	–	2925	+3	CH Stretching	[45]
2364	–	–	2354	+10	OH deformation	[41]
1635	1636	1635	1638	+3	H-O-H bending	[41]
1112	1130	1116	1125	+13	Si-O and/or Al-O stretching vibration	[42]
1023	1023	1024	1027	+4	Si-O-Si symmetric stretch	[40]
776	777	782	779	+3	Al-Mg-OH vibration	[42]
721	–	714	723	+2	Presence of illite	[41]
634	635	637	633	+1	Al-O-Si	[46]
574	572	574	571	-3	Si-O-Al Stretching	[47]
547	545	545	545	-2	Si-O-Si deformation	[48]
469	–	–	–		Si-O deformation	[48]

### 3.2 Surface morphology examination using SEM

**Figure 3:**
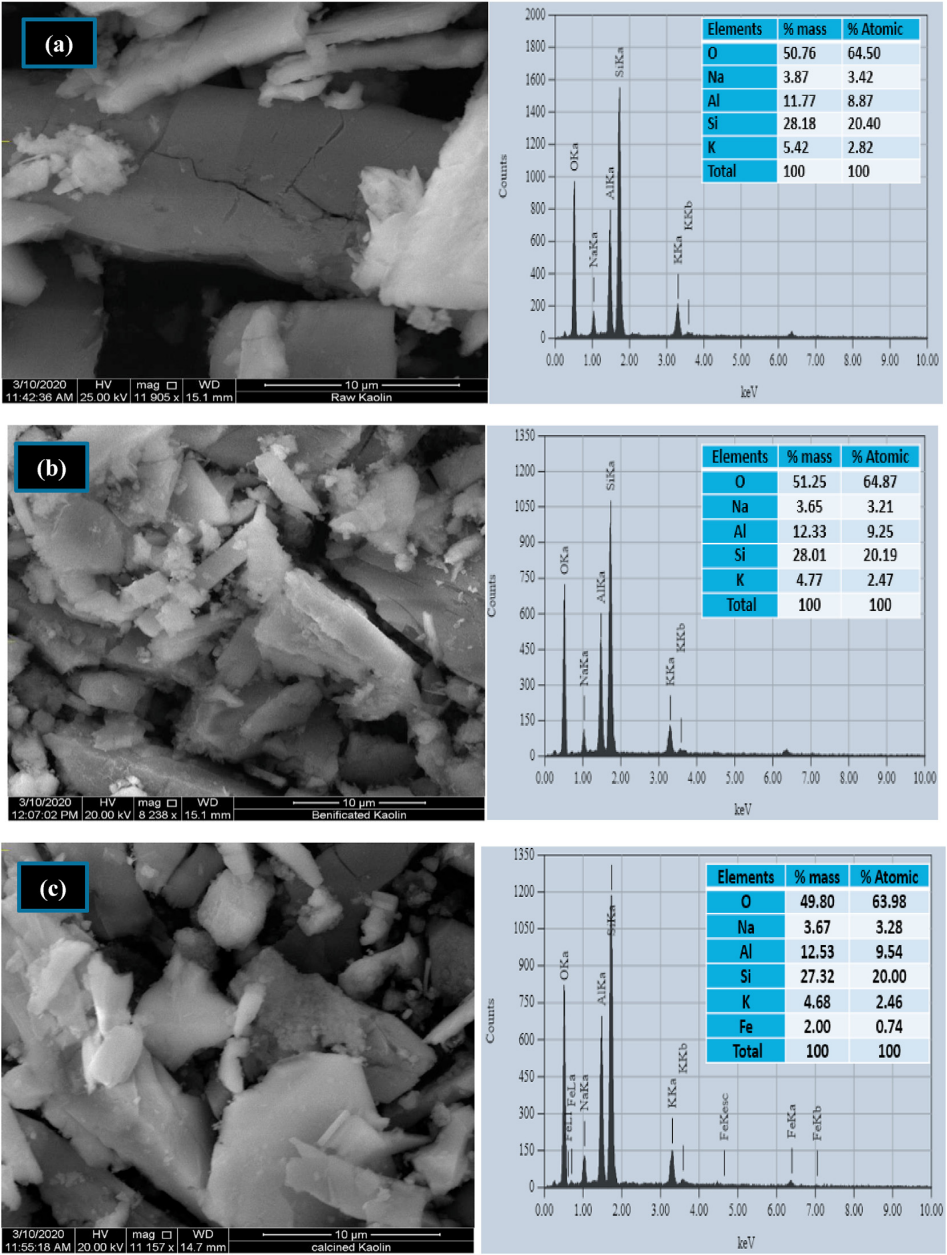
SEM/EDS image of (a) raw (b) beneficiated and (c) calcined kaolin adsorbents.

Scanning Electron Microscopy, SEM was used to examine the surface morphology and the porous nature of raw, beneficiated, and calcined kaolin adsorbents as shown in Figure 3 (a), (b), and (c) respectively which have a direct influence on the adsorption process. The images have unstructured in nature, irregular in shape with uneven edge agglomerate and porous in the surface. The raw kaolin, Figure 3 (a) validate the clay particles of the layered silicates in the form of books tells that the particles are not fully dispersed into individual layers [49, 50]. From the raw kaolin, clear layered rectangular shapes observed an indication that natural kaolinite, without any treatment, is double layer alumino-silicate clay [51]. Besides, adsorptive and porosity nature on the surface is observed and formed a complex structure for beneficiated and calcined kaolin. There is a notable difference in the micrographs obtained for raw and treated (wet and thermal) kaolin adsorbents which are observed more agglomerated and unstructured morphological shapes for the treated kaolin. The particles both for beneficiated and calcined kaolin as shown in Figure 3 (a) and (b) depicted as cuts of paper with many sizes. The adsorptive nature on the surface helps for high adsorption of dye ions from solution [52]. To detect the elemental distributions present in the kaolin powder, Energy Dispersive X-ray Spectroscopy (EDX) was carried out for the three kaolin adsorbents. The major elemental distribution EDX peaks contain silicon, oxygen, aluminum potassium, and sodium for raw and beneficiated kaolin. But iron, as an impurity, is detected in the calcined kaolin. This is due to the embedded iron species in between the two layers (silica and alumina) during calcination intends to expose to the metakaolin surface and can detect with EDX. This is also confirmed that the reddish color has been observed after calcination. Thus, the authors are recommending beneficiations after calcination is important to remove the iron and titanium impurities as per the need of the final product. The mass percentage of Na and K, which considers as an impurity, decreases from 3.87 to 3.63 and 5.42 to 4.77 from raw kaolin to beneficiated kaolin, respectively. This is due to the soluble salts of Na and K were purified in the beneficiation process.

### 3.3 X-ray diffraction studies

**Figure 4:**
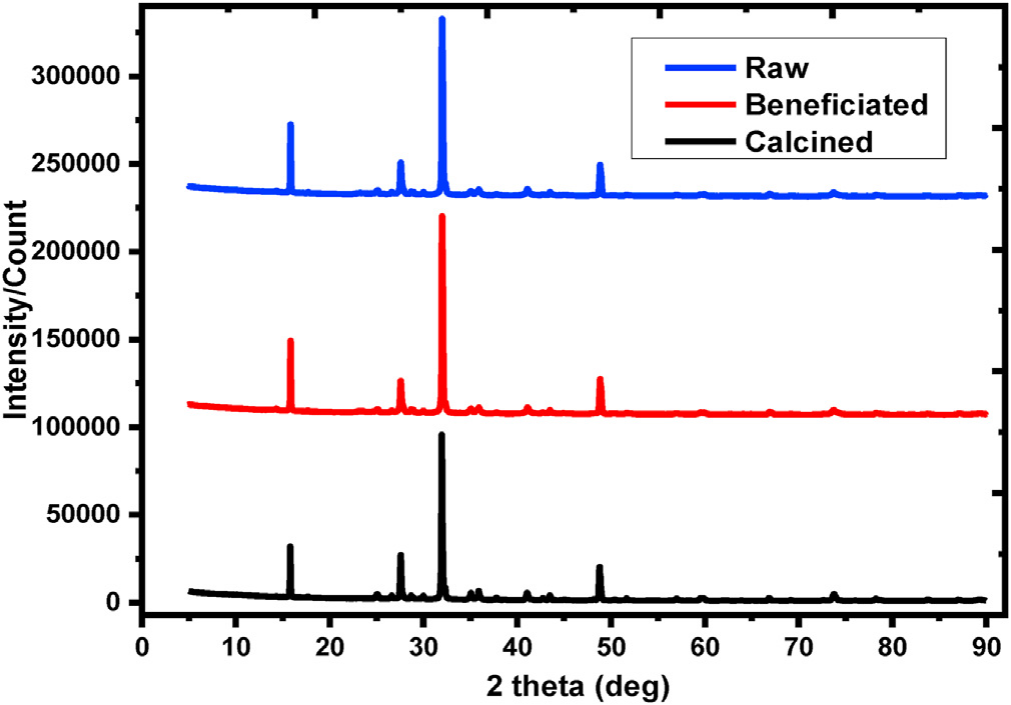
XRD diffraction pattern for raw, beneficiated, and calcined kaolin adsorbents.

X-ray diffraction, XRD, patterns can have a capability to identify the phase change and the crystalline properties of the materials including to determine their amorphous and/or crystals nature. But, kaolin clay is challenging to identify the patterns because of overlapping peaks and chemical interferences contained in kaolinite clay [23, 53]. Dominantly, the diffraction patterns having multiple reflections at 2 θ= 16^0^, 32^0^, and 49^0^ as shown in Figure 4 is a characteristic diffraction pattern for kaolinite clay which has a similar report [24, 25]. A small reflection at 2 θ = 27.5^0^ is evidence on trace quartz which coincides with a similar report with [54]. Slight peak intensity of the kaolin adsorbent was found to increase and the extent of decrease follows the raw >>beneficiated >>calcined which suggests that the kaolin, the sample where taken, is ideal kaolin clay with a crystalline structure and high kaolinite content. A small reflection at 2θ = 74^0^ from the calcined kaolin is an indication of titanium oxide (anatase) which is an impurity released after calcination [55].

### 3.4 Percentage of oxide compositions

The percentage oxide compositions of raw, beneficiated, and calcined were examined as shown in Table 2. The results show the presence of eight elements expressed in percentages in form of their oxides like SiO_2_, Al_2_O_3_, K_2_O, Na_2_O, Fe_2_O_3_, TiO_2_, CaO, MnO, and MgO with its LOI values for raw, beneficiated, and calcined kaolin adsorbents. SiO_2_ and Al_2_O_3_ were the greatest part of kaolin and the result confirms that kaolin is predominantly composed of kaolinite (Al_2_Si_2_O_5_(OH)_4_). It appears that SiO_2_ content is 62.59%, 61.43%, 61.13% with corresponding Al_2_O_3_ 21.5%, 21.11 %, and 20.97% for calcined, raw, and beneficiated respectively. The molar ratio of SiO2/Al2O3 is 2.911, 2.910, and 2.920 for calcined, raw, and beneficiated respectively with 2 for pure kaolinite standard which allows classifying the kaolin clay as a siliceous one [56]. The mass percentage of 3.02% Fe_2_O_3_ is low and is favorable for industrial applications including pharmaceuticals and bioassays due to its high silica and low impurities. The amount of K_2_O (5.63%) and Na_2_O (5.21%), as compared with silica and alumina, for raw kaolin clay suggest that the presence of mica mineral [57]. Also, 0.57 % of TiO_2_ is due to the presence of either rutile or anatase that contained from kaolin clay. Loss on ignition is 2.6% for Ethiopian natural kaolin, where the sample is taken, and this is very far from common values for kaolinite clay-rich materials (14.00%) suggests that the decomposable constituents in the clay too small. Indeed, the present result has given evidence that the high molar ratio of SiO_2_/Al_2_O_3_ (2.92) and the iron oxide amount (3.03%) suggests that the presence of FeO(OH) in the kaolin clay. Some increments of percentage oxide composition, those considered to be as an impurity, in the calcined kaolin are due to surface thermal disintegration of the layers and become exposed to the outer surfaces. Thus wet treatment after calcination is mandatory to separation iron, potassium, and sodium oxides.

**Table 2:** Chemical composition of kaolin adsorbents.

Types of adsorbent	Al_2_O_3_	CaO	Fe_2_O_3_	K_2_O	MgO	MnO	Na_2_O	SiO_2_	TiO_2_	LOI
	%	%	%	%	%	%	%	%	%	%
Raw	21.11	0.45	3.02	5.63	0.15	0.18	5.21	61.43	0.57	2.6
Beneficiated	20.97	0.45	3.02	5.6	0.16	0.18	5.2	61.13	0.57	2.57
Calcined	21.5	0.46	3.13	5.74	0.16	0.19	5.34	62.59	0.59	0.36

### 3.5 Effects of dye concentration, pH, and temperature

**Figure 5:**
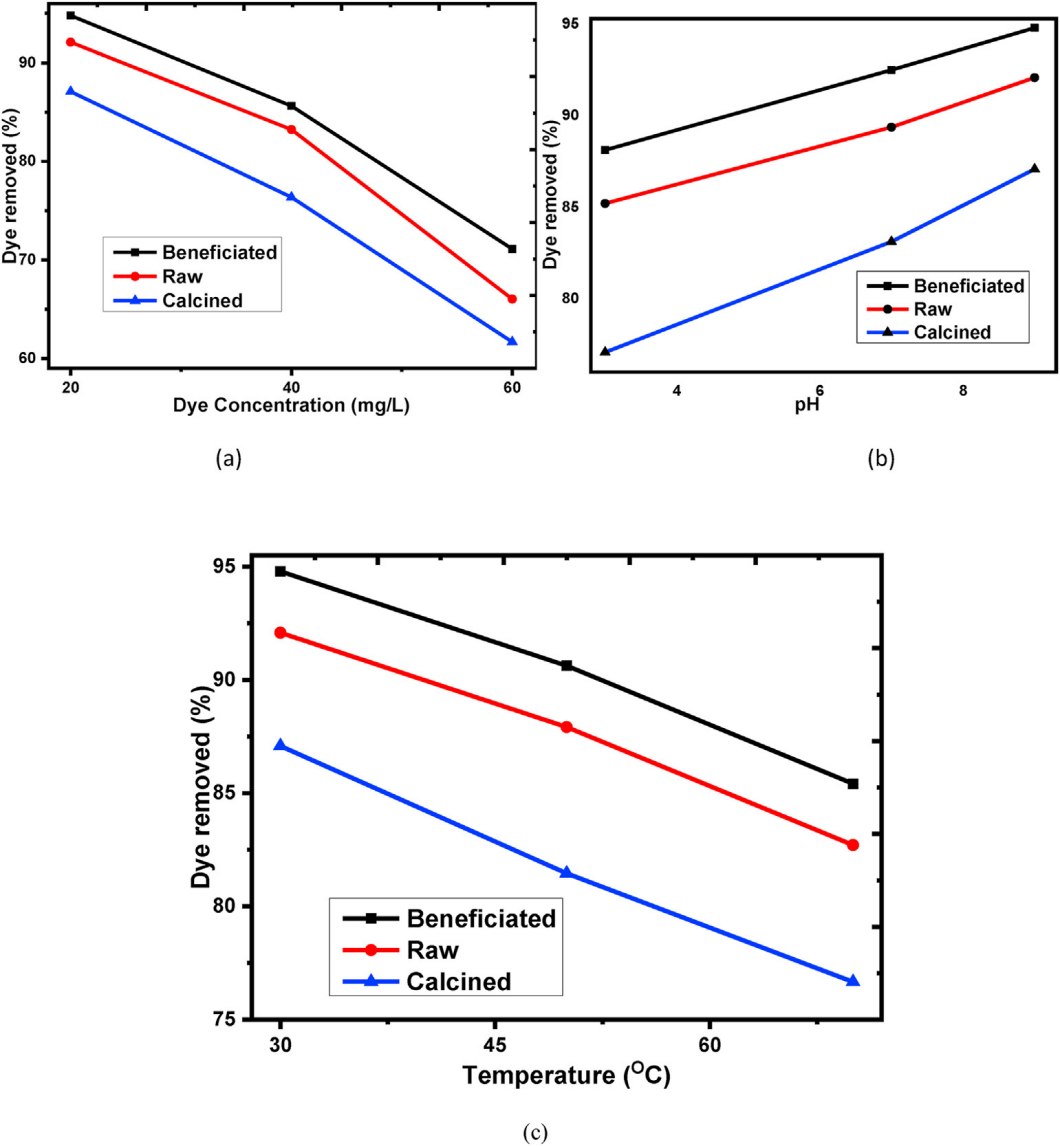
Effect of (a) initial dye concentration, (b) solution pH, (c) temperature for Basic Yellow dye adsorption onto raw, beneficiated, and calcined kaolin adsorbent.

As shown from Figure 5 (a), the percentage removal is decreased from 94.79 to 71.11%, 92.08 to 66.02% & 87.08 to 61.67% for beneficiated, raw, and calcined kaolin adsorbents, respectively as initial dye concentration increases from 20 to 60 mg/L. The maximum dye removal efficiency was obtained at operating conditions of concentration of 20 mg/L, the temperature of 30 °C°C, and solution pH of 9, a contact time of 60 min, and an adsorbent dosage of 1g/100ml. In a similar study, 93.8% of basic yellow dye was removed were achieved with untreated clay at 75 mg/L dye concentration, solution pH of 11, time of 60 min, and adsorbent dosage of 1g/100ml [58]. At low dye concentration, removal efficiency is high. This is due to, the active sites on the surface required for adsorption of the dye molecules are enough. But, as dye concentration increases, the removal efficiency decreases due to the active sites of the adsorbent being occupied and dye molecules re-enter into the liquid phase [59].

The removal efficiency of BY 28 dye is increased from 88.125 -94.77%, 85.20–92.08%, and 77.08–87.08% for the three adsorbents respectively as shown in Figure 5(b) with the increase of solution pH from 3 to 9 at 30 °C°C temperature, 20 mg/L dye concentration, 60 min contact time, and 1g/100ml. The adsorption processes are favored at basic conditions (pH of 9) and this pH condition was used for the other subsequent batch adsorption experiments. Basic condition favorability suggests that BY 28 dye solution carries high hydroxyl groups in the solid-liquid phase which are negatively charged and the surface of the adsorbents is dominantly positively charged makes them for high electrostatic attraction. Conversely, at low pH conditions, more hydrogen ions release into the liquid phase which repulsing with cationic dye and competing in the adsorbent sites being the removal efficiency decreased [60]. A similar report on untreated and acid-treated kaolinite studies as a potential adsorbent was found that basic (cationic) dye removal efficiency is recorded at basic media [58, 61].

The removal efficiency of BY 28 dye is decreased from 94.79 to 85.41%, 92.08 to 82.70%, and 87.08 to 76.67% for the three adsorbents, respectively, as adsorption temperature increases from 30 to 70 °C°C as shown in Figure 5 (c). The experimental condition was constant as a pH of 9, initial dye concentration of 20 mg/L, a contact time of 60 min, and an adsorbent dosage of 1g/100ml. The decrease in removal efficiency with increasing adsorption temperature tells that exothermic conditions in the adsorption process to occur which have a direct relation to the atomic or molecular species tendency to release from the solid surface and dissolves back to the liquid phase [33, 34]. This means that the temperature makes the adsorbent pores expanded and the dye molecules cannot able to retain at the active site. Generally, beneficiated kaolin adsorbent has a slightly higher removal efficiency than raw and calcined kaolin adsorbent for all basic operating conditions.

**Figure 6:**
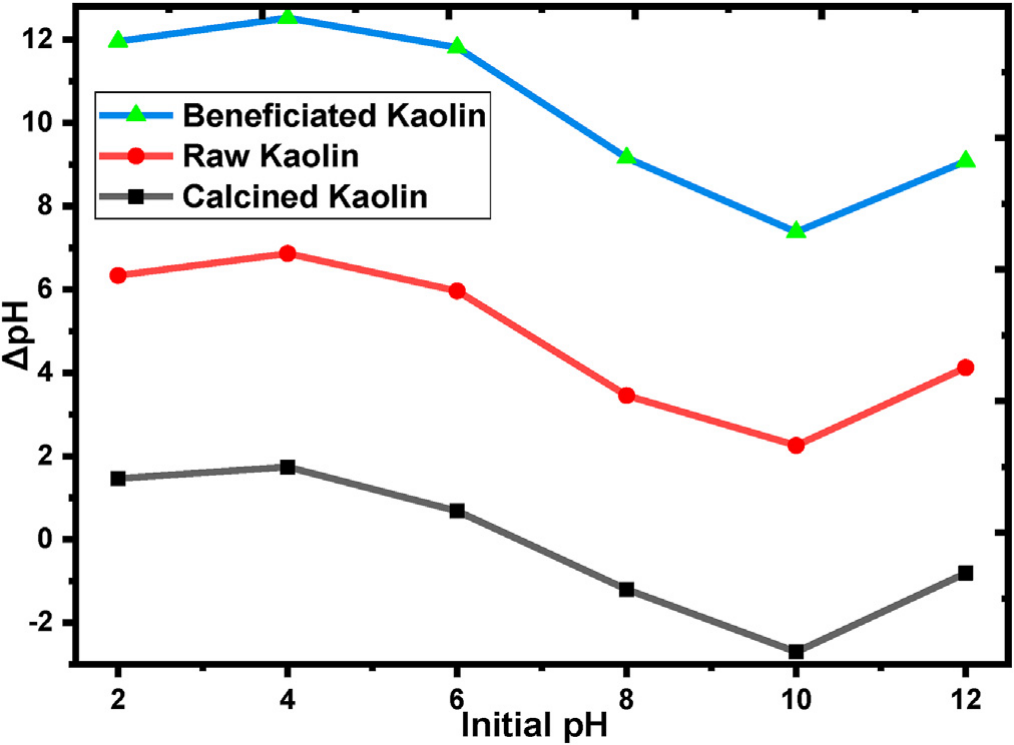
Zero points of charge (ZPC) of raw calcined and beneficiated kaolin adsorbents were used for the BY 28 dye adsorption experiments.

Figure 6 shows that the pHpzc of calcined, raw, and beneficiated kaolin adsorbents. The values of pHpzc found 6.7, 6.9, and 7.2 for calcined, raw powder, and beneficiated kaolin adsorbents, respectively. The zero points of charge signify that the net surface charge of the kaolin adsorbents will be zero [62]. pH values above the zero point of charge the raw as well as treated kaolin adsorbent will be negatively charged but positively charged below the zero points of charge. The highest value of pHpzc was obtained for beneficiated adsorbent due to its surface cleaned up during the beneficiation process [63].

### 3.6 Effects of adsorbent dosage and contact time

**Figure 7:**
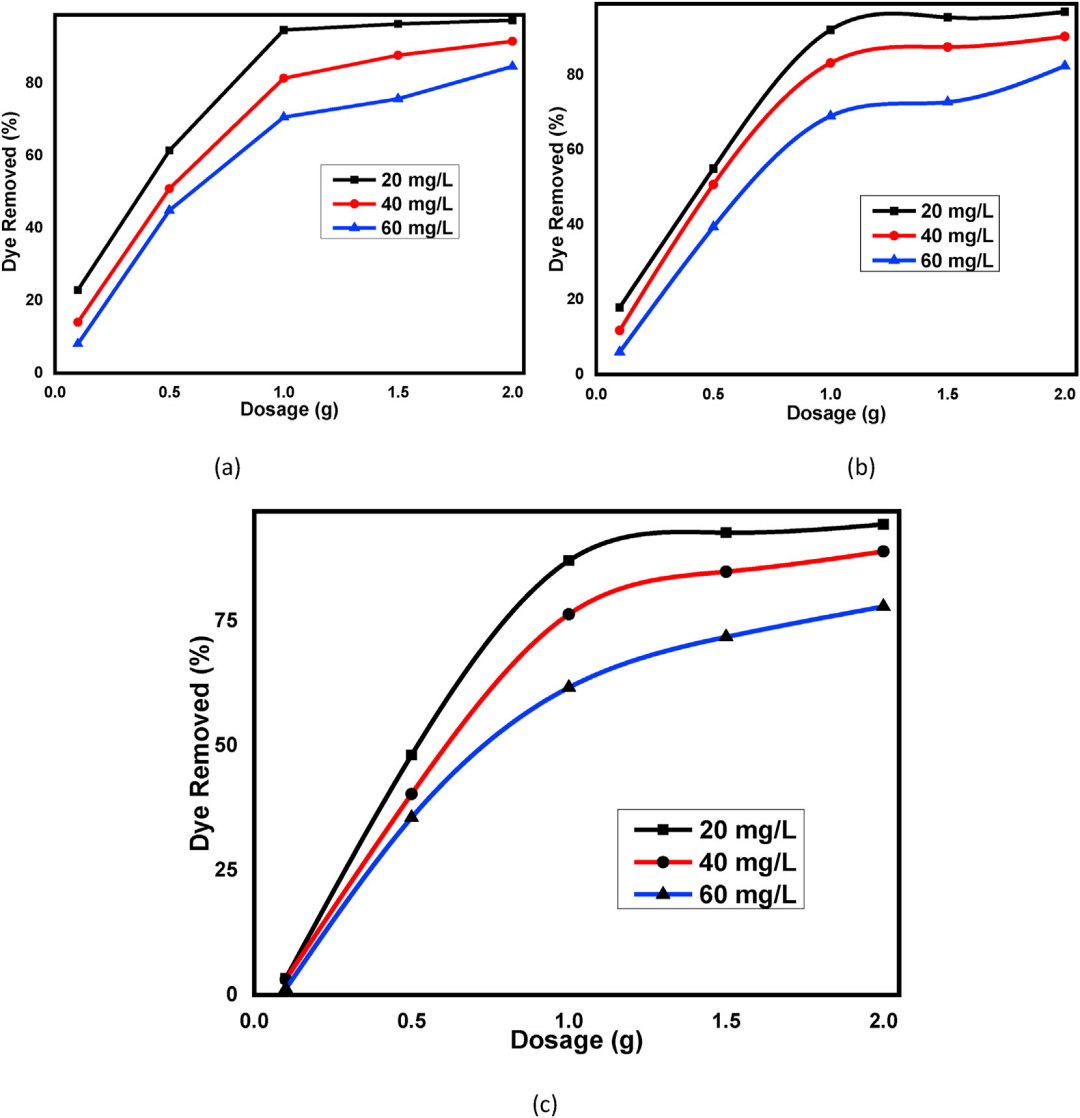
Effect of adsorbent dosage for basic yellow dye adsorption at different initial dye concentration onto (a) Beneficiated, (b) Raw and (c) Calcined kaolin adsorbents.

As shown in Figure 7 (a), (b), and (c) the removal efficiency of BY 28 dye is increased from 22.92 to 97.5%, 17.92–96.87%, and 3.33–94.38% for the three adsorbents, respectively with adsorbent dosage increases from 0.1 g to 2 g. Solution pH, temperature, contact time were fixed as 9, 30°C 60 min respectively for different dye concentrations (20 mg/L, 40 mg/L, and 60 mg/L). Increasing adsorbent dosage increases removal efficiency of BY 28 dye suggests that attribution of available sorption surface and more adsorption sites can occur [64]. From 0.1 to 1.0 g of for the three kaolin adsorbents, a dramatic increase in removal efficiency was observed. But from 1.0 g to the 2.0g, the removal efficiency becomes constant even for the higher dye concentration (60 mg/L) and suggests that 1.0 g adsorbent is enough for the above experimental conditions. 97.5% removal efficacy of methylene blue dye, a cationic dye, from aqueous solution using kaolin adsorbent with the same operating conditions were reported by Kaur et al. [65].

**Figure 8:**
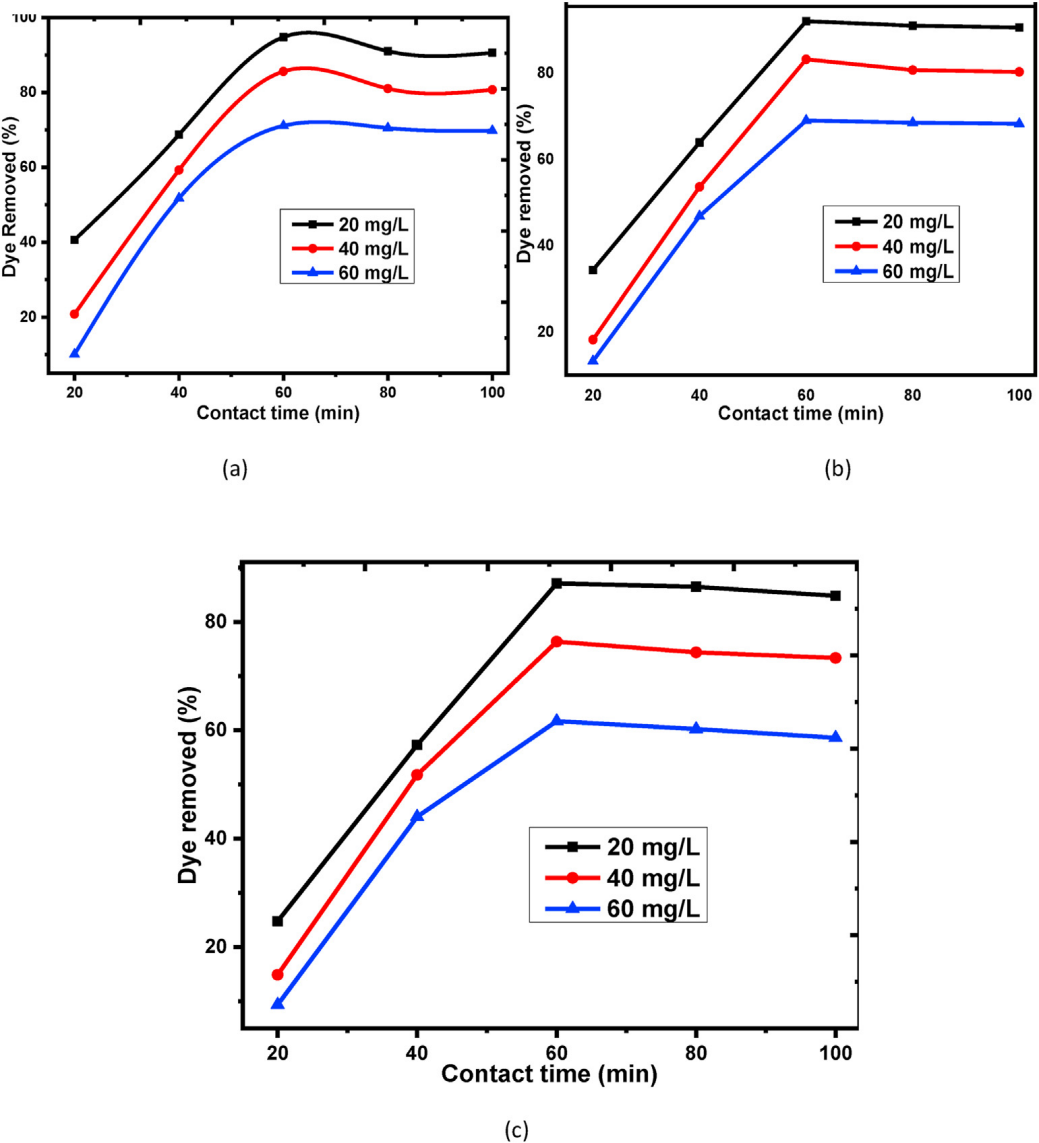
Effect of contact time for basic yellow dye adsorption at different initial dye concentration onto (a) Beneficiated, (b) Raw and (c) Calcined kaolin adsorbents.

For the evaluations of the exact time required for high percentage removal of BY 28 dye adsorption onto the raw and treated adsorbents, contact time is very important. To obtain the maximum removal efficiency of BY 28 dye, five points (20–100 min) with 20 min increment were taken at 1g/100mL dosage, 9 solutions pH, and temperature of 30 °C with different (20, 40 and 60 mg/L) initial dye concentrations. As can be seen, the removal efficiency recorded as a maximum removal of 94.76%, 83.23% and 70.69% onto beneficiated; 92.08%, 81.46% and 69.02 onto raw; and 87.08%,76.35% and 61.67% onto calcined kaolin adsorbents, respectively for initial dye concentration of 20 mg/L, 40 mg/L and 60 mg/L. As can be seen from Figure 8: (a), (b), and (c), the removal efficiency of BY 28 dye is dramatically increasing as increasing in contact time up to 60 min. Further, an increase up to 100 min has not shown increases in the percentage removal efficiency in the adsorption process due to adsorption processes have its own specific time based on its adsorption kinetics at given operation parameters [66]. Thus, it can be elucidated that 60 min is the equilibrium time at the above conditions. A similar report of the equilibrium time at 60 min was recorded with the same experimental condition in the study of the adsorption process of methyl orange dye onto mesoporous carbon material [67].

### 3.7 Thermodynamic study

Thermodynamic parameters of standard free Gibbs energy change ($\Delta {\text{G}}^{0}$), standard enthalpy change ($\Delta {\text{H}}^{0}$), and standard entropy change ($\Delta {\text{S}}^{0}$) were calculated to examine the spontaneity behaviors in the adsorption process for raw calcined and beneficiated kaolin adsorbent. The thermodynamic parameters have been calculated from equilibrium concentrations and different temperature profiles [68] and ΔG0 were calculated from Eqs. (9) and (10).

**Figure 9:**
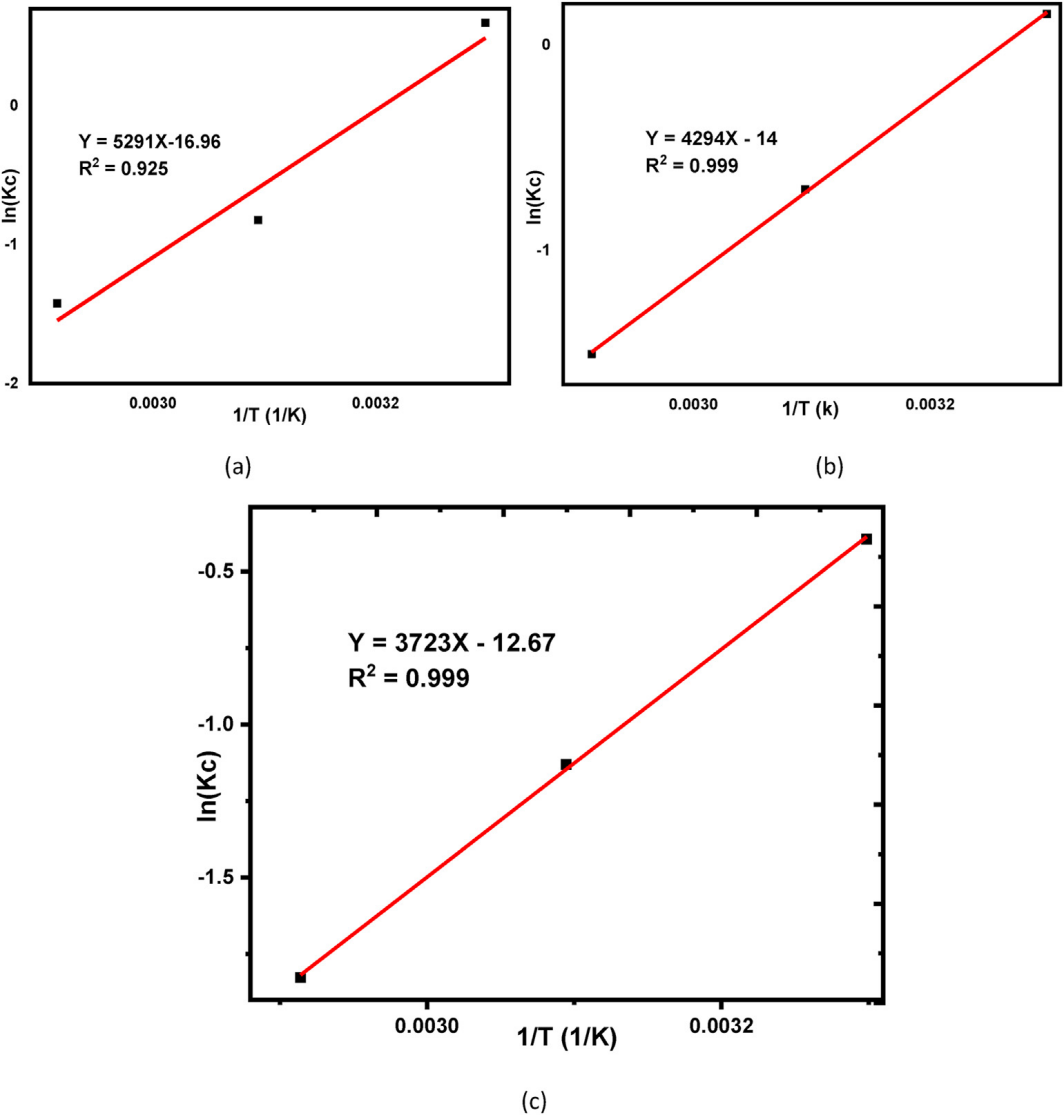
Thermodynamic study for Basic Yellow dye adsorption over (a) Beneficiated, (b) Raw, and (c) Calcined kaolin adsorbents.

The values of ΔH° (KJ/mol) and ΔS° were calculated from the slope and intercept of a linear plot ln Kc versus 1/T of beneficiated, raw, and calcined kaolin respectively shown in Figure 9 (a), (b), and (c). Also, the values of ΔG° were calculated from ΔH° and ΔS°, and all thermodynamic parameters are shown in Table 3.

**Table 3:** Thermodynamic parameters for beneficiated, raw, and calcined kaolin adsorbent.

Types of kaolin adsorbents	$\Delta G^{0}$ [KJ/mol]			$\Delta H^{0}$[KJ/mol]	$\Delta S^{0}$[KJ/mol]
	Temperature [K]				
	303.15	323.15	343.15		
Beneficiated	-1.243	1.576	4.396	-43.989	-0.141
Raw	-0.414	1.913	4.240	-35.700	-0.116
Calcined	0.980	3.087	5.193	-30.953	-0.105

From the thermodynamic parameters shown in Table 3, at the lowest temperature (303.15K) the values of $\Delta {\text{G}}^{0}$ have recorded a minimum value of -1.243 kJ/mol, -0.414 kJ/mol, and 0.980 kJ/mol for beneficiated, raw, and calcined adsorbent respectively. A small increase in $\Delta {\text{G}}^{0}$ value from -1.243, -0.414, and 0.980 for beneficiated, raw, and calcined respectively tells that the high adsorption of BY 28 dye has occurred with beneficiated > calcined > raw adsorbents. This suggests that the thermodynamic behavior in the adsorption process at the lowest temperature is more feasible and spontaneous as compared with the higher temperature one which coincides with studying the effect of temperature for removal efficiency [69]. Also, the negative values of ΔH^o^ and ΔS° suggest that the adsorption phenomenon is exothermic and the process is enthalpy driven respectively [70, 71]. The enthalpy driven correlates with the degree of randomness at the solid-liquid interface which increases adsorptions of BY 28 dye onto the adsorbent.

### 3.8 Adsorption isotherms

Langmuir and Freundlich isotherm models were employed for the adsorption study of BY 28 dye onto raw, beneficiated, and calcined kaolin adsorbents. Adsorption isotherm models are used to describe the equilibrium behaviors of adsorbate adsorbed by using certain adsorbent materials. Moreover, studying the adsorption isotherm models is one of the important factors in the description of the adsorption systems [72] based on solid-liquid interaction in the adsorption process [73].

**Figure 10:**
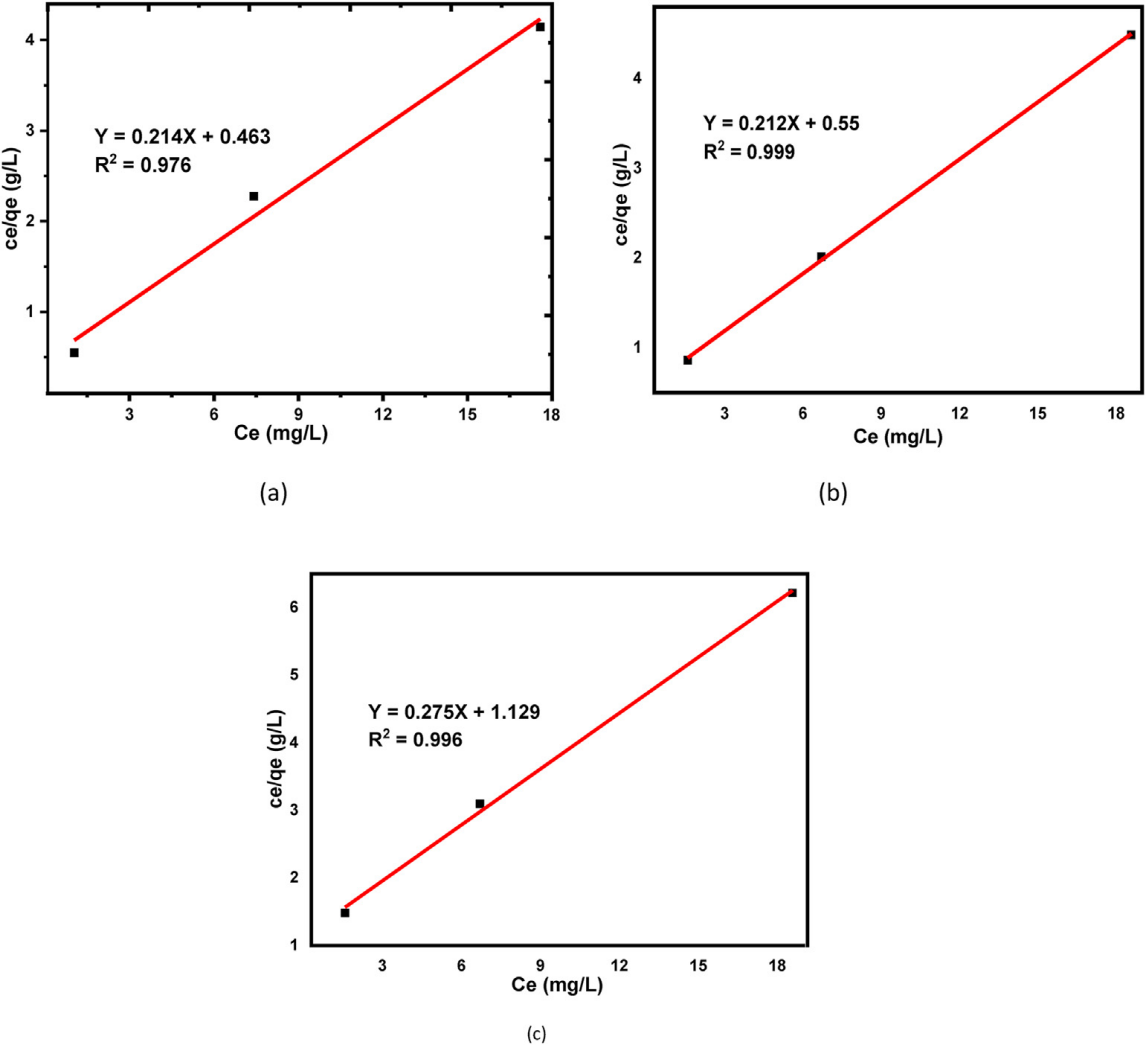
Langmuir isotherm for basic yellow dye removal onto (a) Beneficiated (b) Raw and (c) Calcined kaolin adsorbents.

The principle of the Langmuir isotherm model describes the adsorbate can be adsorbed as a monolayer from the homogeneous adsorbent materials [72]. In this adsorption experiment, the Langmuir isotherm model equation is used to understand the degree of BY 28 dye adsorption tendency onto kaolin adsorbents [72, 74]. Thus, equilibrium data of adsorbates in the solid-liquid phase were analyzed with the Langmuir model equation as shown in Eq. (4). R^2^ values from the Langmuir model are; 0.999, 0.996, and 0.978 for raw, calcined, and beneficiated kaolin adsorbents respectively as shown in Table 3. The higher value for raw kaolin as compared with calcined and beneficiated one tells that dominantly a monolayer adsorption process occurs and the adsorbent is homogeneous suggests that all the adsorbed BY 28 dye are in contact a chemisorption processes [75]. The calculated values of a separation factor, R_L,_ as from Eq. (6) for beneficiated, raw and calcined adsorbents were 7.55 × 10^−3^, 6.38 × 10^−3,^ and 4.04 × 10^−3^ respectively suggest that the adsorption process is favorable. Also, the positive correlation was confirmed from the Langmuir isotherm model as shown in Figure 10 (a), (b), and (c) for beneficiated, raw and calcined kaolin adsorbents, respectively.

**Figure 11:**
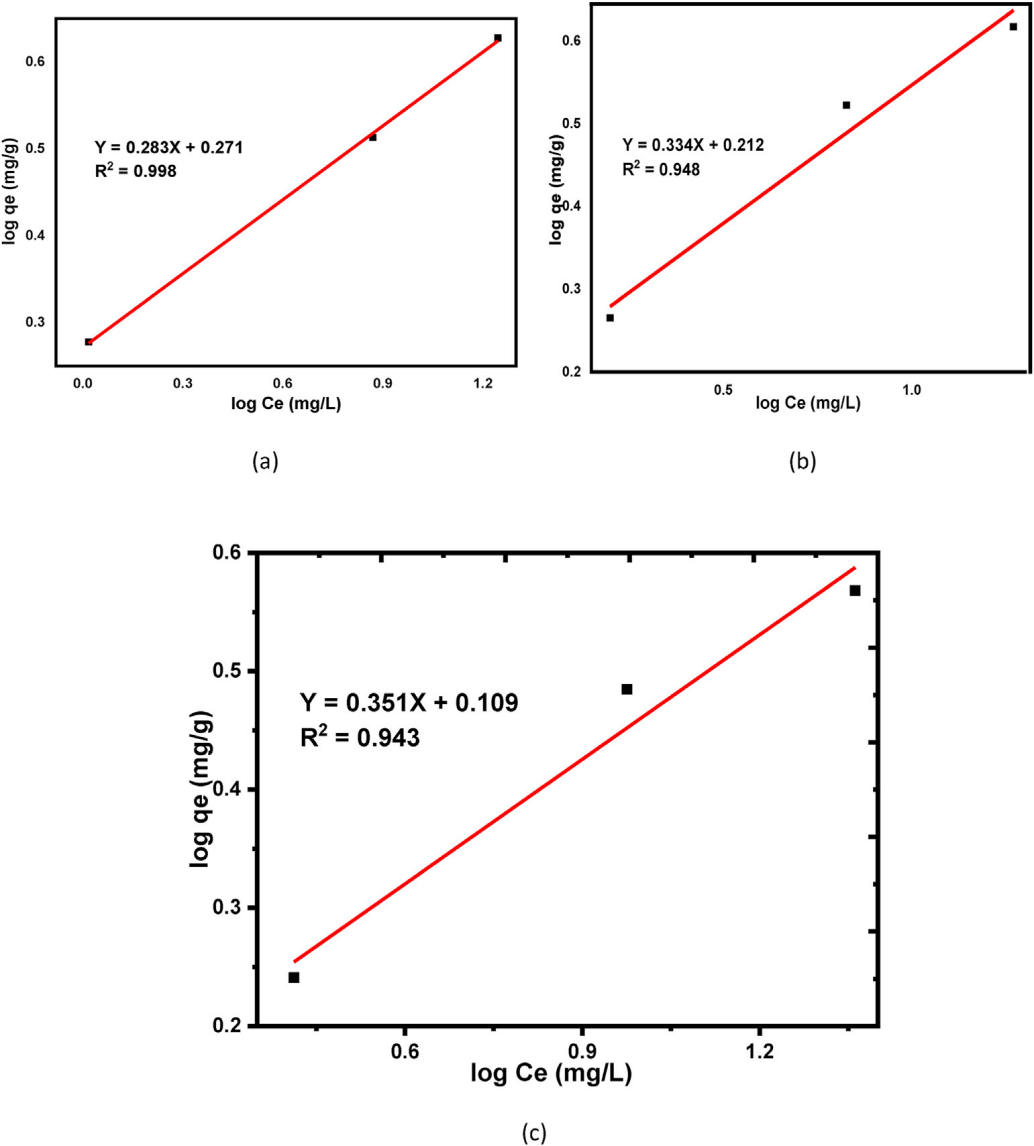
Freundlich isotherm for basic yellow dye removal onto (a) Beneficiated (b) Raw and (c) Calcined kaolin adsorbents.

Freundlich isotherm adsorption model is an empirical equation that determines the heterogeneous adsorption phenomenon on the adsorbent surface as shown from linearized equation Eq. (5) [76, 77]. The value of sorption intensity (n) can tell us the favorability of the adsorption processes as n = 2 to 10 represent well, n = 1 to 2 moderately difficult, and n = less than 1 poor adsorption characteristics [76]. The values of sorption intensity (n) were found as 3.571, 2.994, and 2.849, as shown in Table 4 for beneficiated, raw, and calcined kaolin adsorbents, suggests that the favorability of adsorption is well fitted for the three adsorbents and were good for beneficiated kaolin as compared with raw and calcined adsorbents. The values of regression coefficients (R^2^) are higher for beneficiated kaolin in Freundlich isotherm models as shown in Table 4 tells that the adsorption processes are well fitted for beneficiated kaolin adsorbent but Langmuir is well fitted for raw kaolin adsorbent. This indicates that dominantly a multilayer adsorption (physical sorption) process occurs suggests that the adsorption space accommodates more than one layer of molecules and not all adsorbed BY 28 dyes are in contact with the surface layer of the adsorbent but also intra-surface adsorption takes place. Also, the linear fitting of the Freundlich isotherm model is shown in Figure 11 (a), (b), and (c) for beneficiated, raw and calcined kaolin adsorbents, respectively.

**Table 4:** Langmuir and Freundlich isotherm parameters for for basic yellow dye adsorption.

Constants	Langmuir Model			Freundlich Model		
	Beneficiated	Raw	Calcined	Beneficiated	Raw	Calcined
q_m_ (mg/g)	2.174	1.818	0.885	–	–	–
K_L_ (L/mg)	2.190	2.594	4.105	–	–	–
K_f_ (mg/g)	–	–	–	1.309	1.236	1.115
n	–	–	–	3.571	2.994	2.849
R^2^	0.978	0.999	0.996	0.998	0.948	0.943

The adsorption capacity depends on adsorbate concentration and adsorbate-adsorbent ratio; quality and amount of active cite on the; propensity of the solute adsorbed compared to the residue. Also, the adsorption processes depend on the solute nature which has a response on their electro-statistic nature, the hydration energy, and affinity. In the adsorptions process, adsorbate binding approaches to the adsorbent is important. So, low adsorption capacity does not mean that removal efficiency will be low based mentioned aspects [78].

### 3.9 Adsorption kinetics

To design adsorption unit operations in the wastewater treatment plant, the rate at which the adsorbates can be removed from homogenous effluent is very important [67]. Two common adsorption kinetics models, pseudo-first and second-order equations were used as shown in Eqs. (7) and (8), respectively. This is important to validate the experimental data with the predicted model values with regression coefficients (R^2^) express. A relatively higher R2 value from the predicted model tells that their applicability in the adsorption processes. The pseudo-first-order kinetics correlation coefficient is calculated as 0.764, 0.730, 0.886 for beneficiated, raw, and calcined kaolin adsorbents respectively, and 0.901, 0.826, and 0.397 for pseudo-second-order as shown in Table 4.

**Figure 12:**
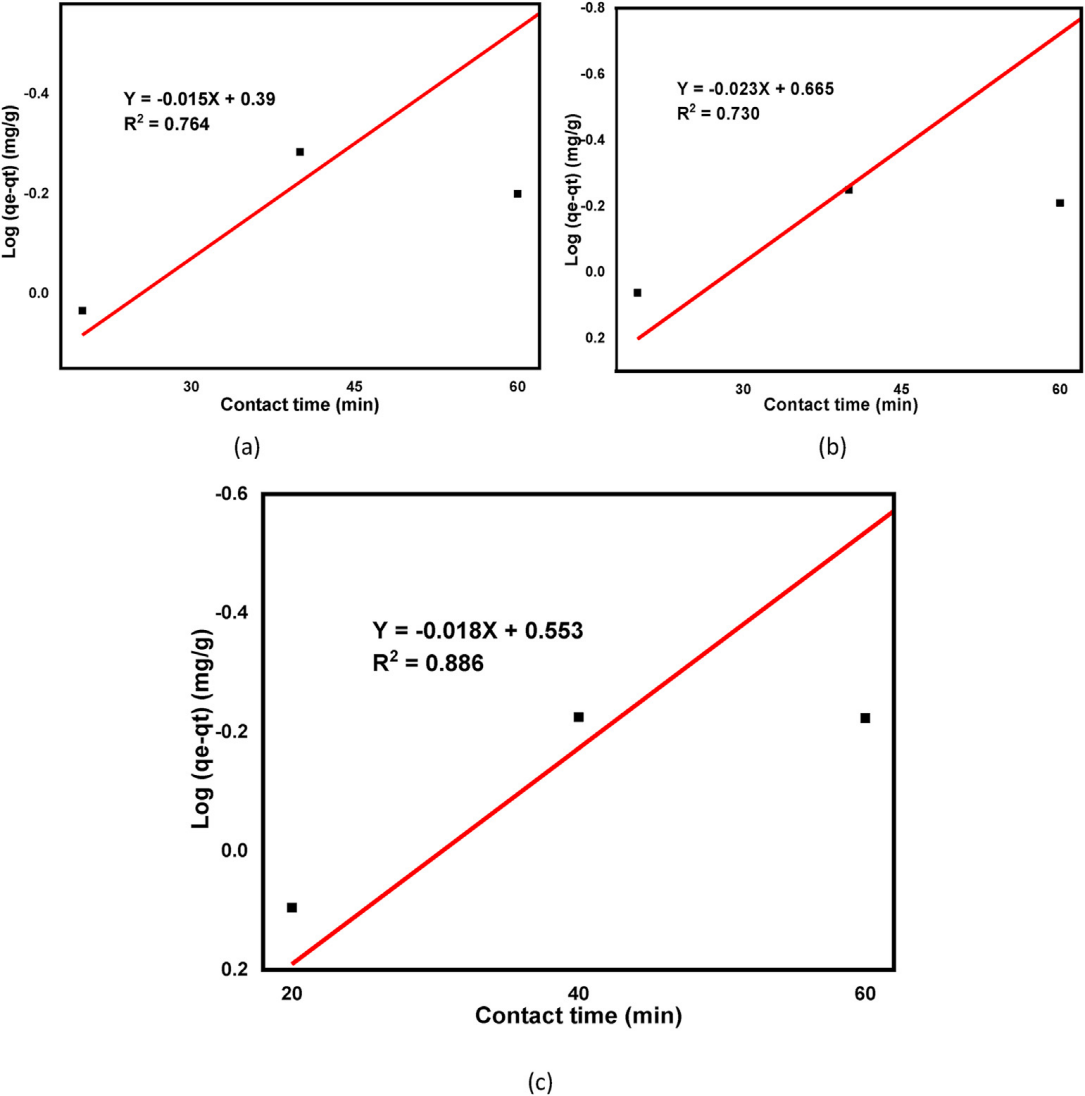
Pseudo first order for adsorption of basic yellow dye onto (a) Beneficiated, (b) Raw and (c) Calcined kaolin adsorbents.

**Figure 13:**
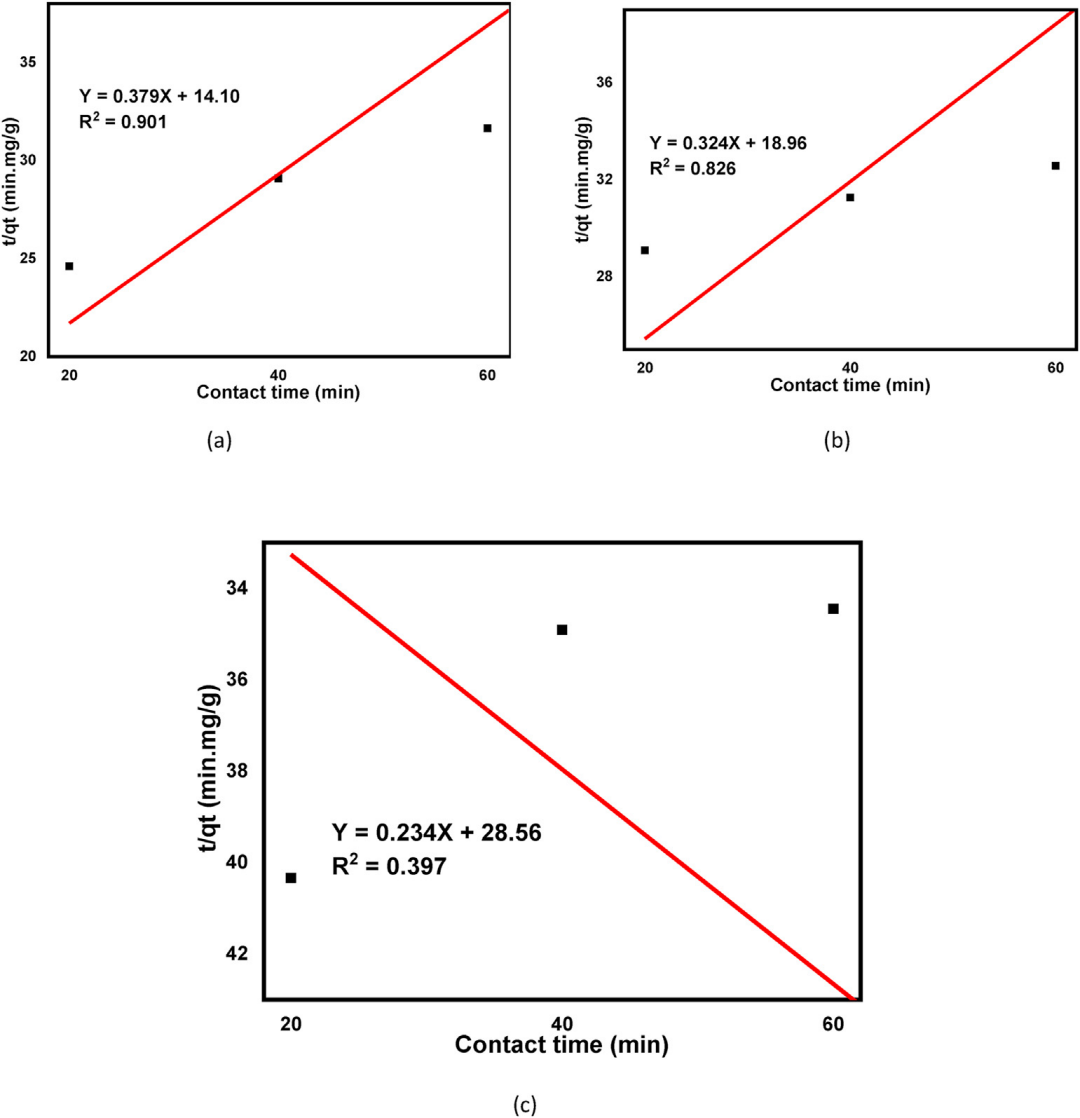
Pseudo second order for adsorption of basic yellow dye onto (a) Beneficiated, (b), Raw and (c) Calcined kaolin adsorbents.

The R^2^ value follows the pseudo-second-order for beneficiated and raw kaolin adsorbents, but, it follows a pseudo-first-order kinetic model for calcined kaolin adsorbent as shown in Table 5. The R^2^ value of pseudo-second and first-order was calculated as 0.901 and 0.764 respectively for beneficiated kaolin adsorbent as shown in Table 5. From this, it can be deduced that the adsorption phenomenon is subject more with a chemical adsorption process in the solid-liquid phases [31, 79] but dominated with physical adsorption for the calcined kaolin adsorbent. The linear fitting plots for both the pseudo-first and second-order kinetics model is shown in Figures 12 and 13 for beneficiated, raw, and calcined kaolin adsorbents respectively which showed that a positive linear fitting except that the calcined kaolin in the pseudo-second-order kinetics. Similar reports were recorded for arsenic removals using guava leaf biomass, mango bark, and bagasse as an adsorbent [80, 81].

**Table 5:** Pseudo first and second-order model parameters for adsorption of basic yellow dye.

Constants	Pseudo First Order Model			Pseudo Second-Order Model		
	Beneficiated	Raw	Calcined	Beneficiated	Raw	Calcined
qe, exp.(mg/g)	1.896	1.842	1.742	1.896	1.842	1.742
qe (mg/g)	2.418	2.027	1.991	2.632	3.086	4.111
K_1_ (min^−1^)	-0.048	0.053	0.051	–	–	–
K_2_ (g/mg∗min)	–	–	–	0.010	0.005	0.0028
R^2^	0.764	0.730	0.886	0.901	0.826	0.397

### 3.10 Regeneration and reuse of adsorbent

**Figure 14:**
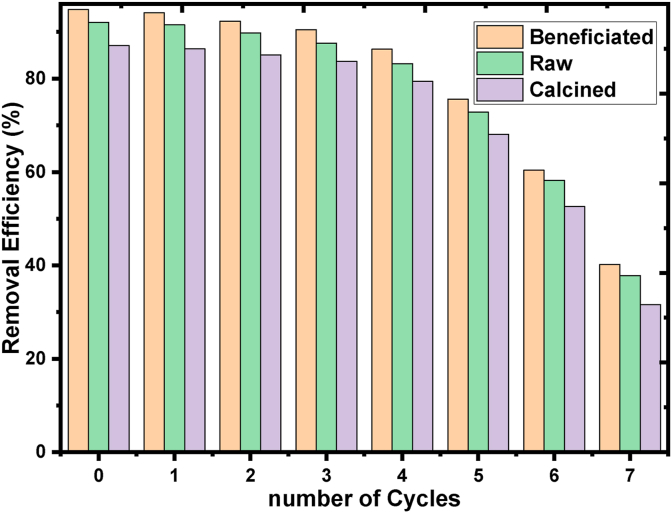
Basic Yellow (BY28) Dye removal efficiency (%) with Beneficiated, Raw and Calcined Kaolin Adsorbent as a function of regeneration cycles.

To use the adsorbents in the pilot and real scale, it would be practical and economical. So, the adsorbents having high sorption capacities after the number of recycling is important because of the amount of adsorbent necessary for the treatment of dye can be used in multiple times. Thus, cost-effective raw and treated kaolin adsorbents for BY28 dye removal from the wastewater should be desorbed and reused. To assess the regeneration potential of BY28 dye adsorbed of the three adsorbents were, seven subsequent adsorption-desorption cycles were conducted using 0.1 M K_2_CO_3_ chemical. The percentage removal efficiency of beneficiated, raw, and calcined kaolin adsorbent is decreased with continuous regeneration from 94.97, 92.08, and 87.08 %, respectively to 40.2, 37.8, and 31.6%, respectively at 7^th^ cycle of reuse as shown in Figure 14. The sorption efficacy for the first 5^th^ cycle 75.6, 72.8, and 68.1%) is an effective and dramatic decrease in the 6th and 7th cycles. The decrease in sorption removal efficiency of recycled adsorbents is due to not enough actives sites on the surface of the adsorbent which were diminished electrostatic and regenerating agents sintering effect [82].

### 3.11 Sorbents adsorption capacity comparison

Different types of basic (cationic) dye (Basic blue 9, Malachite Green, Methylene Blue, Crystal violet, etc …). Adsorption capacities were reported using various adsorbents [83]. Especially, methylene blue was studied extensively. Since molecular size, structure, and color index value of the dyes has their effect on the adsorption capacity of the sorbent, the present authors assessed only the basic yellow dye to compare the adsorption capacity on different sorbents. A comparative analysis of sorbent capacities for the adsorption of basic dyes is illustrated in Table 6. The adsorption capacities of the adsorbent are dependent on operating parameters. Thus, the sorption capacities are listed with the corresponding basic operation parameters (solution pH, dye concentration, and sorbent dosage).

**Table 6:** A Maximum adsorption capacity of basic yellow dye on to various sorbents with specified experimental conditions (initial dye concentration, pH, and sorbent dose).

Sorbent type	qm (mg/g)	Operating parameters			Reference
		pH	Conc. (mg/L)	Sorbent dose (g/L)	
reed	140	6	100	0.5	[84]
coal-based granular activated carbon	3.8285	9	150	0.8	[85]
Seaweed	4.7	5	25	5	[86]
conch shells	34.08	11	25	6	[87]
calcined mussel shells	14.62	10	50	1	[88]
Calcined eggshells	28.87	10	60	2	[89]
clinoptilolite	8.7	6.5	15	0.75	[90]
Green algae	27	9	50	0.5	[91]
Tea waste	43.88	6	100	20	[92]
Moroccan Clay	60	12	300	30	[93]
Natural untreated clay	76.92	11	80	2	[17]
activated carbon (apricot stones)	77.7	10	16	5	[94]
Kaolin (beneficiated)	1.896	9	20	1	Present study

## 4 Conclusion

Utilization of natural resources, especially clay material, in the country for the development of adsorbent for dye purification is an alternative and cost-effective. Adsorbents were effectively synthesized from Ethiopian for the removal of basic yellow 28 dye from the aqueous solution. The chemical compositions of raw and prepared kaolin adsorbents confirm that silicon and aluminum oxides are contained dominantly as per the required standard of pure kaolin which has a big role to be an adsorbent.

The raw and treated kaolin adsorbent were examined for effective removals of basic yellow 28 dye. During the adsorption process in the batch experimental study, all tested basic operation parameters have a significant effect on the removal efficiencies of basic yellow 28 dye for raw as well as treated kaolin. Morphological examination using SEM a clear layered rectangular shape were identified in the raw kaolin and disappeared after calcination ma and formed as book layer features. Also, Alumina and silica elemental distribution is mapped with EDS analysis. The XRD diffraction patterns suggest that a crystalline kaolinite clay showed. The optimum conditions of initial dye concentration of 20 mg/L, solution pH of 9, adsorbent dosage 1g/100 mL, the temperature of 30 °C and contact time of 60 min around 94% removal efficiency were obtained. The adsorption kinetics is fitted with pseudo-second-order models for the beneficiated and raw kaolin adsorbents but follows the pseudo-first-order model for calcined kaolin adsorbent. Finding out non-conventional adsorbents for dye wastewater treatment is attracting attention. Due to the sustainable alternative resources, the use of kaolin materials for dye removal is cost-effective. Ended, Ethiopian kaolin can be used as a potential adsorbent for dye wastewater treatment. As can be seen from chemical compositions, metal oxide other than silicon and aluminum oxides increases for the calcined kaolin. Thus, the authors would like to recommend than beneficiation after calcination is important to remove those metals which are considered as impurities.

## Declarations

### Author contribution statement

Tadele A. Aragaw: Conceived and designed the experiments; Analyzed and interpreted the data; Contributed reagents, materials, analysis tools or data; Wrote the paper.

Fikiru T. Angerasa: Performed the experiments; Contributed reagents, materials, analysis tools or data.

### Funding statement

This research did not receive any specific grant from funding agencies in the public, commercial, or not-for-profit sectors.

### Competing interest statement

The authors declare no conflict of interest.

### Additional information

No additional information is available for this paper.
